# On-Line Raman Spectroscopic Study of Cytochromes’ Redox State of Biofilms in Microbial Fuel Cells

**DOI:** 10.3390/molecules24030646

**Published:** 2019-02-12

**Authors:** Adolf Krige, Magnus Sjöblom, Kerstin Ramser, Paul Christakopoulos, Ulrika Rova

**Affiliations:** 1 Biochemical Process Engineering, Division of Chemical Engineering, Department of Civil, Environmental and Natural Resources Engineering, Luleå University of Technology, SE-971 87 Luleå, Sweden; adolf.krige@ltu.se (A.K.); paul.christakopoulos@ltu.se (P.C.); Ulrika.Rova@ltu.se (U.R.); 2 Experimental Mechanics, Division of Fluid and Experimental Mechanics, Department of Engineering Sciences and Mathematics, Luleå University of Technology, SE-971 87 Luleå, Sweden; kerstin.ramser@ltu.se

**Keywords:** microbial fuel cell, Raman spectroscopy, *Geobactersulfurreducens*, cytochrome-C, Omc

## Abstract

Bio-electrochemical systems such as microbial fuel cells and microbial electrosynthesis cells depend on efficient electron transfer between the microorganisms and the electrodes. Understanding the mechanisms and dynamics of the electron transfer is important in order to design more efficient reactors, as well as modifying microorganisms for enhanced electricity production. *Geobacter* are well known for their ability to form thick biofilms and transfer electrons to the surfaces of electrodes. Currently, there are not many “on-line” systems for monitoring the activity of the biofilm and the electron transfer process without harming the biofilm. Raman microscopy was shown to be capable of providing biochemical information, i.e., the redox state of C-type cytochromes, which is integral to external electron transfer, without harming the biofilm. In the current study, a custom 3D printed flow-through cuvette was used in order to analyze the oxidation state of the C-type cytochromes of suspended cultures of three *Geobacter sulfurreducens* strains (PCA, KN400 and ΔpilA). It was found that the oxidation state is a good indicator of the metabolic state of the cells. Furthermore, an anaerobic fluidic system enabling in situ Raman measurements was designed and applied successfully to monitor and characterize *G. sulfurreducens* biofilms during electricity generation, for both a wild strain, PCA, and a mutant, ΔS. The cytochrome redox state, monitored by the Raman peak areas, could be modulated by applying different poise voltages to the electrodes. This also correlated with the modulation of current transferred from the cytochromes to the electrode. The Raman peak area changed in a predictable and reversible manner, indicating that the system could be used for analyzing the oxidation state of the proteins responsible for the electron transfer process and the kinetics thereof in-situ.

## 1. Introduction

Electroactive biofilms have long been known to be capable of generating electrical power through extracellular electron transfer, converting the chemical energy found in chemical bonds of organic compounds. Microbial fuel cells (MFCs) have shown promise in wastewater treatment [1], bioremediation [2] or even as an alternative renewable energy source in remote areas [3]. In MFCs, under anaerobic conditions, catalytic reactions of microorganisms oxidize an electron donor through extracellular electron transfer to the anode. The cathode is then exposed to an electron acceptor to facilitate an electrical current [4,5]. The Gram-negative bacteria from the *Geobacter* genus are considered to be well-known electroactive bacteria, although several other micro-organisms show some electro-activity [6].

Efficient electron transfer between the microorganisms and electrodes is a key factor for the efficiency improvement of bio-electrochemical systems, such as MFCs and microbial electrosynthesis cells. Understanding the mechanisms and dynamics of the electron transfer is important in order to design electrodes in a more efficient way, as well as modifying microorganisms in an attempt to increase the current density [7]. This is evident when one looks at the improvements already achieved using improved electrode designs by, for example, the addition of Mn^4+^ or Fe^3+^ to graphite electrodes or the increase of the specific surface area [8,9].

*Geobacter sulfurreducens* is well known for its ability to form thick biofilms and transfer electrons to the surfaces of electrodes without the need for mediators [10,11]. Electrons are generated from the oxidation of NADH, which is derived from organic matter oxidation, in association with the proton pumping required for energy production. The subsequent electron transfer serves merely to dispose of the electrons.

In this study three abiotic electron acceptors were used, i.e., fumarate and Fe(III)oxide for suspended cultures and a poised graphite electrode for an on-line MFC system. Fumarate is an intra-cellular electron acceptor constituting a relatively simple electron transport chain and its role in respiration has been extensively studied [12,13]. Furthermore, it has been shown that *G. sulfurreducens* can neither oxidize fumarate nor use fumarate as a carbon or energy source since the succinate produced from formate reduction is not oxidized in the tri-carboxylic acid (TCA) cycle but excreted into the medium [14].

**Figure 1:**
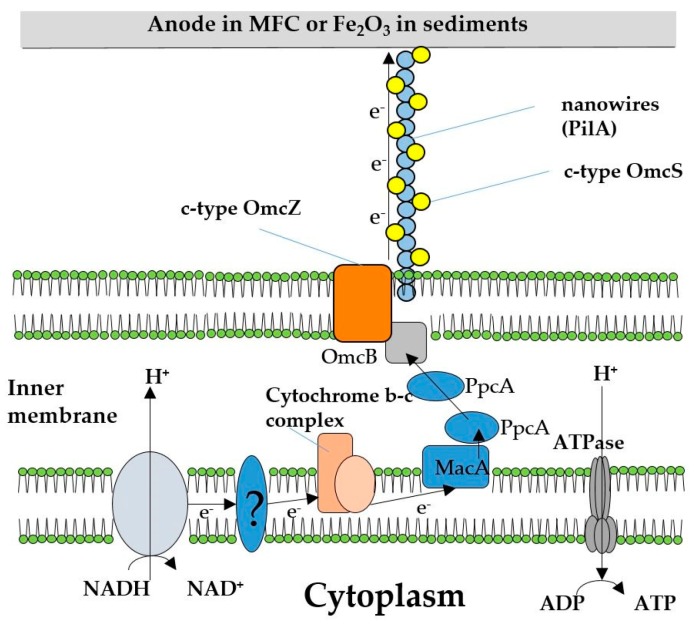
A schematic for the mechanism for extracellular electron transfer of *Geobacter sulfurreducens* (Adapted from [7,17]).

Insoluble Fe(III)oxide is a well-known electron acceptor for *G. sulfurreducens* and also the predominant form of Fe(III) in most soils and sediments. Under Fe(III)-reducing conditions, the TCA cycle is operated as a closed loop, producing eight electrons per molecule of acetate oxidized. In contrast to the use of fumarate as an electron acceptor, electrons are transported outside the cell, leaving protons in the cytoplasm. It is unlikely that this final electron transfer yields energy to the cell [7]. Translocation of these protons dissipates the membrane potential and acidifies the cytoplasm, which is theoretically the reason why growth rates are approximately 3 fold lower during Fe(III) reduction [15]. Several enzymes participate in the electron transport chain, including several outer membrane C-type cytochromes (OMC) (namely OmcS, OmcZ and OmcB, as seen in Figure 1) [7,16].

In order to learn more about the electron transfer process, in-situ surveillance is a necessity. Several techniques have been used in an attempt to characterize the biofilm and shed light on the mechanisms and dynamics involved in electron transfer. These methods do however each have their own unique advantages and disadvantages, and most require the removal of the biofilm from an anaerobic reactor to an aerobic environment, thus oxidizing the cytochromes. Some of which are discussed below:

Confocal Laser Scanning Microscopy can provide quite a lot of information, both on the biofilm structure and on the microbial activity or interaction (for example when stained with a live-dead stain [18]). However, most stains are toxic and kill the biofilm, therefore continuous measurements are not possible [19]. Using fluorescence in situ hybridization, which uses fluorescently labeled DNA-probes, specific DNA sequences can be targeted, which can be used to, for example, show interspecies electron transfer [20,21]. This method is, however, also not suited for continuous measurements. Electrochemical Impedance Spectroscopy (EIS) offers a non-destructive tool to analyze both the electrode interface and the electrochemical reactions involved, yet this gives limited information as to the internal cellular mechanisms [22]. DNA extraction and sequencing can be useful when analyzing biofilms grown from a consortium, yet it requires invasive methods such as the removal of parts of the biofilm or sections of the electrode [23,24]. Optical microscopy can also be used to obtain information on the localization of the biofilm, yet it is limited as to the amount of information that can be obtained [25]. MFCs are complex bio-electrochemical systems, and would therefore require more than a single technique to be completely understood.

C-type cytochromes are known to be good Raman scatterers, especially when a resonance excitation wavelength is chosen, and the sheer abundance of C-type cytochromes makes the cultures/biofilms visibly red. The genome of *G. sulfurreducens* contains 111 putative genes coding for C-type cytochromes, several of them being multi-heme cytochromes [26]. Four strong bands have previously been observed in the Raman spectra of *G. sulfurreducens* at 747, 1133, 1310 and 1583 cm^−1^, which can be ascribed to the excitation of the heme groups of cytochrome-c that are prevalent in the biofilms [27,28]. This also compares well with cytochrome c from horse heart [29]. The cytochromes have also been shown to function as capacitors for the cells, storing electrons under some environmental conditions [30,31].

Four mutants of *G. sulfurreducens* were used, in order to get specific information about the electron transfer to extracellular and intracellular electron acceptors, i.e., the wild strain PCA [32], KN400, a strain with enhanced capacity for current production developed from PCA through selective pressure (this enhanced capacity was associated with a greater abundance of electrically conductive pili) [33], a strain lacking PilA (ΔpilA), the structural pilin protein which have been implicated in long-range electron transfer through anode biofilms, and a strain lacking the cytochrome OmcS that is localized on the pili (ΔOmcS), which is believed to be important in Fe(III) oxide reduction.

The aim of this paper was to evaluate a non-invasive method to analyze the biofilm of an MFC while the system is still active and electrically poised. Currently there are not many “on-line” systems for monitoring the activity of the biofilm and the electron transfer process without harming the biofilm [31,34]. By measuring the spectroscopic properties of active and poised biofilms (specifically, the Raman fingerprint OMCs), it would be possible to measure the effect that poising the cell at different potentials has on the oxidation state of different biofilms. Because the OMCs are directly involved in the electron transfer system, and the cells require electron transfer for growth, Raman spectra can give information on the metabolic state of the biofilm, as well as how stable the biofilm is, depending on the selected strain. This could also be combined with the use of different mutants in order to gain insight into specific electron transfer mechanisms.

In the current study, an anaerobic fluidic system enabling in situ Raman measurements was designed and applied to monitor and characterize *Geobacter* biofilms during electricity generation.

## 2. Results and Discussion

In order to obtain preliminary Raman measurements, experiments were carried out using cells suspended in a custom-made 3D printed flow-through cuvette. Secondly cells were grown in a chemostat, in order to obtain a repeatable sample in the exponential phase, and measurements were taken after resuspension in a buffer. Finally, biofilms were grown in a stack microbial fuel cell and measurements were taken while the cell was poised at different voltages. Raman spectra were analyzed as described in Section 3.5.

### 2.1. Suspended Cells Raman Measurements

Three strains were used during the initial tests, namely KN400, PCA and ΔpilA. These strains were selected in order to observe the effect that the level of abundance of pili have on the oxidative state of OMCs. During these experiments, Raman measurements and analysis of metabolites were done at different time points during batch cultivation of *Geobacter* strains, where the Raman measurements were started once significant biomass was obtained. During the Raman measurements using different strains of *G. sulfurreducens*, very little difference was observed in how the strains reacted to the applied stimuli, showing proportionally similar decreases/increases in Raman peak area when, for example, fumarate was added.

**Figure 2:**
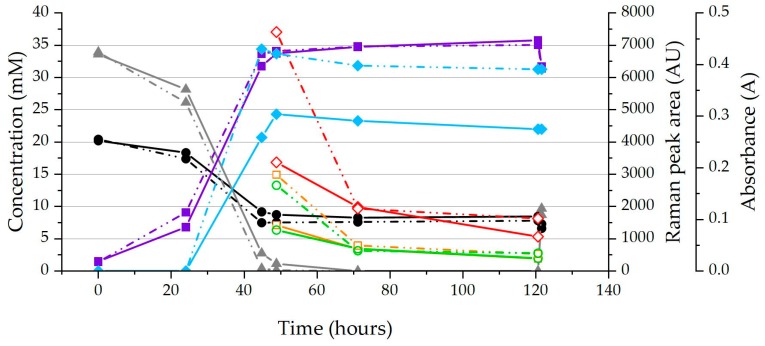
Raman peak areas (Raman peak areas: 1314 cm^−1^ ○, 1130 cm^−1^ □, 749 cm^−1^ ◇), metabolite concentrations (Fumarate ▲, Succinate ■ and Acetate ●) and OD600 (Absorbance ◆) of ΔpilA and KN400 (KN400 is shown with a solid line and ΔpilA with dotted line) of an example run showing the significant difference between maximum Raman peak areas of the strains, as well as the significant decrease as stationary phase is reached.

Figure 2 shows an example of the metabolite concentrations, as well as the absorbance (i.e., OD600, a common method of measuring cell concentration [35]) and the Raman peak areas (measured when enough cell mass was obtained) for the strains ΔpilA and KN400 without the addition of supplementary fumarate. The production and excretion of succinate into the medium during growth on acetate and fumarate is consistent with previous metabolic studies on *G. sulfurreducens* [15,36]. For all the strains tested, the peak area dropped 4.1 ± 0.83 fold when the culture entered the stationary phase (i.e., when a substrate has been completely consumed, fumarate in this case, and the primary metabolism ceases, around 70 h in this case), suggesting that the peaks are related to primary metabolism and not only to the total biomass.

**Figure 3:**
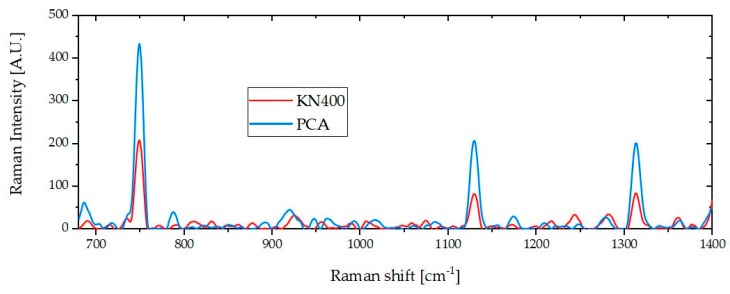
Processed Raman spectra of suspended cultures, showing an example of the difference between PCA and KN400, which is related to the cytochrome-c quantity and oxidation state. Both samples had an OD600 of 0.24.

Despite the similar trends observed in the Raman peak areas from the different strains, the maximum peak area obtained was significantly lower for KN400 than for that of PCA and ΔpilA (as can be seen in Figure 3). For example, for the measured Raman peak area shown in Figure 3 the area at 747, 1133 and 1310 cm^−1^ for KN400 were on average 39% that of the same peaks for PCA, despite the fact that the cell concentration was the same for each sample. The KN400 strain was specifically grown to be electroactive, yet in a previous study C-type cytochromes were found to be much less abundant in KN400, compared to the PCA strain [33], which would fit with the results obtained in the present study.

The drop in Raman peak area, when the stationary phase was reached, could be explained by the capacitor hypothesis [31] where the cytochromes are charged during growth on the rich medium where high amounts of acetate (electron donor) and fumarate (electron acceptor) are present, resulting in a capacitor-like electron “storage system”. It is well known that fumarate reduction is coupled to NADPH and NADH oxidation and the reduced equivalents delivered into the electron transport chain.

Since the system is dynamic, there is a continuous charge and discharge of the cytochromes. When the fumarate is exhausted the primary metabolism stops (even though acetate is still present (6–7 mM)) resulting in the cessation of the charging (reduction) of the cytochromes and a decrease in the peak area.

**Figure 4:**
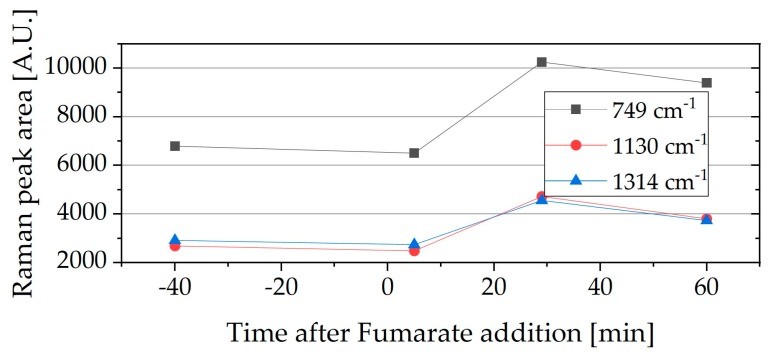
Raman peak area of one ΔpilA sample where fumarate was almost consumed and after the addition of additional fumarate (Raman peak areas: 1314 cm^−1^ ▲, 1130 cm^−1^ ●, 749 cm^−1^ ■), (1.5 mM fumarate, 5.4% of initial, and 10 mM acetate, 50 h into the cultivation).

When the fumarate was close to being depleted (1–6% of initial concentration) and additional electron acceptor was added, an increase in the peak area was detected, indicating that there was an increase in metabolic activity that resulted in the eventual charging of the cytochromes. This increase is typically slightly delayed, occurring only after some minutes. Figure 4 shows a typical example of this, where fumarate was added to a sample in which the fumarate was close to being depleted (5.4% of the initial).

### 2.2. Chemostat Grown Biomass Raman Measurements

**Figure 5:**
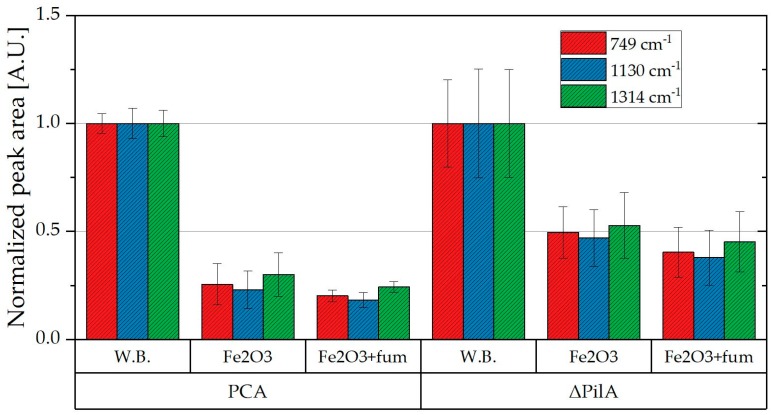
Normalized Raman peak areas of PCA and ΔpilA samples grown in a chemostat, suspended in a wash buffer (W.B.) and the same samples after the sequential addition of Fe(III)oxide and fumarate. (The error bars show the standard deviation of 6–9 Raman measurements).

Two strains of *G. sulfurreducens* (PCA and ΔpilA) were grown in chemostats in order to observe the electron transfer without the conductive PilA to an external electron acceptor (Fe(III)oxide) and also to compare that to an electron acceptor that can be internalized, namely fumarate. The cells were centrifuged and resuspended in wash buffer, without an electron acceptor or donor. KN400 was not selected because of the significant differences in OMC expression. The results were normalized with respect to the average peak areas of the sample suspended only in wash buffer (13,725; 6375 and 5930 for ΔpilA and 17,560; 8075 and 7655 for PCA, of the peaks at 749 cm^−1^; 1130 cm^−1^ and 1314 cm^−1^ respectively) as seen in Figure 5. There was a 50–80% decrease in the peak area when Fe(III) oxide was added. This shows that the mechanisms involved in the electron transfer to an external source does not require pili to effectively oxidize the OMCs.

This might be due to direct transfer using OmcZ which has been found to participate in homogeneous electron transfer (through the biofilm bulk) [37]. Furthermore, the decrease in the peak area is much more significant for PCA than for ΔpilA, suggesting a higher capacity for electron transport in the wild type when compared to ΔpilA. This is consistent with the presence of the conductive pili of the PCA strain which are used to reduce external electron acceptors such as Fe(III) oxide.

### 2.3. Stack-MFC Raman Measurements

By using stack-MFC, it was demonstrated that we can modulate the redox state of the C-type cytochromes, which is reflected in the Raman peak area, by controlling the poising potential. The tests were done using two strains, the wild type PCA and a mutant ΔOmcS. ΔOmcS was used instead of the ΔpilA strain used above, since ΔpilA does not form electro active biofilms.

**Figure 6:**
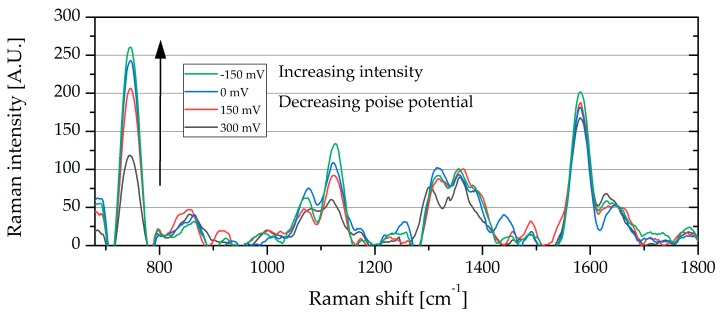
Processed Raman spectra showing the changes in the strong peak at 745 cm^−1^, which is related to the cytochrome oxidation state.

**Figure 7:**
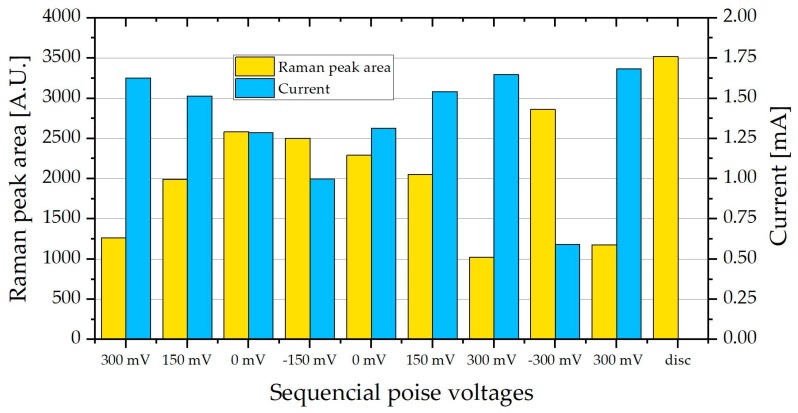
The Raman peak area at 745 cm^−1^ of PCA biofilm in a microbial fuel cell (MFC) stack, average of two measurements along with the current produced at the different poised level. The current is inversely related to the peak area.

From Figure 6 the Raman peaks that showed the largest relative change in peak area are the peaks at 747 and 1133 cm^−1^, whereas the peaks at 1310 and 1583 cm^−1^ were smaller and showed less change at the different poised levels. The area under the peak at 747 cm^−1^ was calculated for each spectrum at the different poised values, from spectra like that seen in Figure 6. A clear increase in peak area is observed in Figure 7, as the poised potential decreases, followed by a decrease as the poised potential is then increased again. Similarly, a larger increase in peak area is observed when the cell is disconnected completely.

The clear increase in peak area when the resistance to current is increased shows the build-up of a charge in the cytochromes i.e., the reduction of the cytochromes. Subsequently, when the cell is poised at +300 mV the resistance is at its lowest, leading to the lowest peak area, and the oxidation of cytochromes. This is very similar to the fluorescence response observed by Núñez [31], where the fluorescence was lost when the cytochrome was oxidized.

**Figure 8:**
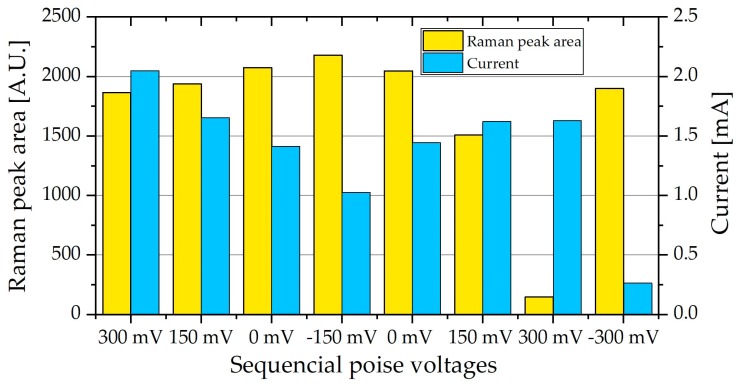
The Raman peak area at 745 cm^−1^ of ∆OmcS biofilm in an MFC stack, average of two measurements, as well as the current produced at the different poised levels. It can be seen that the current is inversely related to the peak area. A decrease in expected current as well as the expected peak areas can be seen at the last three poise levels.

The measurements were repeated using a ΔOmcS biofilm. The ΔOmcS mutant does not carry the OmcS protein located on the pilA nanowires. Although it was initially thought that OmcS is important for the conductivity of the pilA nanowires, studies have shown that the presence of OmcS on pili is not sufficient to confer conductivity to pili [38]. It was, however, not possible to achieve as thick a biofilm as when using PCA. However, as seen in Figure 8, a similar peak area was achieved during the measurements. The biofilm showed a similar response, with the peak area increasing as the current and poise voltage decreased.

However, the peak area decreased drastically when the biofilm was poised at 300 mV for the second time and the current was slightly lower than that of the first time the cell was poised at 300 mV. Since the current represents the activity of the entire biofilm it does suggest a slight decrease in the biofilm activity. The decrease in Raman peak area is, however, much more drastic than the decrease in current and we believe that this is a combination of the biofilm on the measurement spot being affected by the light source, as well as a general decrease in biofilm activity. This, however, requires further investigation, possibly a decrease in Raman laser power or exposure time. The general decrease can also be seen in the fact that current does not increase back to the original values as in Figure 8. The results do, however, show that *G. sulfurreducens* does not require OmcS for electron transfer to an external electrode.

## 3. Materials and Methods

### 3.1. Preparation of Measurement Cells

**Figure 9:**
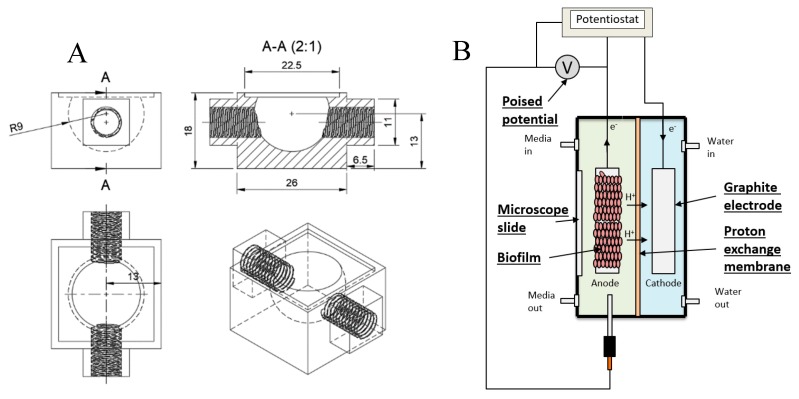
Schematics of (**A**) the 3D printed cell and (**B**) the Microbial fuel cell used for Raman measurements.

3D Printed cell: A flow-through cuvette was designed and printed in a black PETG plastic using a standard 3D printer (Prusa i3 MK3, Praha, Czech Republic). The cell had dimensions of 26 × 26 mm and 18 mm high with an 18 mm diameter cut-sphere as the containment area, with a total volume of 2.5 mL excluding the connecting tubes. A cover-slide was then glued to the cuvette using a silicone sealant. A schematic can be seen in Figure 9.

Stack microbial fuel cell: A two-chamber stack reactor was designed and built (Mercury engineering, Edenvale, South Africa) using polycarbonate for the main body and cover plates, with stainless-steel tube-fittings and silicone gaskets. The two chambers were separated with a proton exchange membrane (Nafion N117), each fitted with solid graphite electrodes, and the anode chamber was fitted with an Ag/AgCl reference electrode. A three-electrode configuration setup was used, consisting of two graphite cuboids (70 × 22 × 10 mm) serving as working and counter electrodes and an Ag-AgCl reference electrode. A window was cut out of one cover plate in order to fit a microscope slide directly over the anode.

### 3.2. Microbes, Media and Inoculation

*Geobacter sulfurreducens* strain PCA (ATCC 51573, DSMZ 12127), as well as modified versions of the strain (ΔOmcS, ΔOmcZ, ΔPilA and KN400), obtained from Dr. Ashley Franks, La Trobe University, Bundoora, Australia, was used in all studies. The strains ΔOmcS and ΔOmcZ have the genes for the outer membrane cytochromes S and Z removed, whereas ΔPilA has the the pilA gene removed. KN400 on the other hand is a variant of *G. sulfurreducens* with enhanced capacity for current production [33].

*G. sulfurreducens* inoculums were grown in a slightly modified NBAF media (pH of 6.8), with acetate (10 mM) and fumarate (40 mM) as the electron donor and acceptor, respectively [39]. The base composition of NBAF per liter of deionized water is 0.42 g of KH_2_PO_4_, 0.22 g of K_2_HPO_4_, 0.2 g of NH_4_Cl, 0.38 g of KCl, 0.36 g of NaCl, 0.03 g of CaCl_2_, 0.1 g of MgSO_4_·7H_2_O, 1.8 g of NaHCO_3_, 0.43 g of Na_2_CO_3_, 1.0 mL of 100 mM Na_2_SeO_4_, 10.0 mL of a vitamin solution, and 10.0 mL of NB trace mineral solution. The composition of the NB trace mineral solution per liter of deionized water is 2.14 g of nitriloacetic acid, 0.1 g of MnCl_2_·4H_2_O, 0.3 g of FeSO_4_·7H_2_O, 0.17 g of CoCl_2_·6H_2_O, 0.2 g of ZnSO_4_·7H_2_O, 0.03 g of CuCl_2_ 2H_2_O, 0.005 g of AlK(SO_4_) _2_ 12H_2_O, 0.005 g of H_3_BO_3_, 0.09 g of Na_2_MoO_4_, 0.11 g of NiSO_4_ 6H_2_O, and 0.02 g of N_2_WO_4_ 2H_2_O [40].

The composition of the vitamin solution per liter of deionized water is 0.002 g of Biotin, 0.005 g of Pantothenic Acid, 0.0001 g of B-12, 0.005 g of p-aminobenzoic acid, 0.005 g of Thioctic Acid, 0.005 g of Nicotinic Acid, 0.005 g of Thiamine, 0.005 g of Riboflavin, 0.01 g of Pyridoxine HCl and 0.002 g of Folic Acid [41].

In order to ensure that the electron acceptor is limiting, for the suspended cell Raman measurements, 15 mL of a 100 mM sodium acetate solution was added to 100 mL NBAF media. This resulted in a starting concentration of approximately 20 mM acetate and 32 mM fumarate after a 10% inoculation.

The microbial fuel cell stack was started with a 10% inoculation of a freshwater media, containing 20 mM acetate and 40 mM fumarate, as previously described [42]. After batch operation, the anode chamber was fed continuously using the freshwater media containing 10 mM acetate and no fumarate at a flow rate of 0.5 mL min^−1^.

### 3.3. 3-D printed Cuvette Suspended Cell Raman Measurements

**Figure 10:**
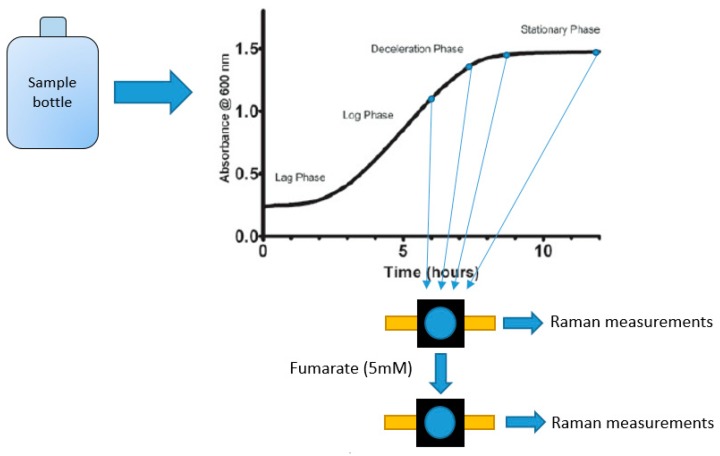
Schematic of the sampling process used for suspended cell Raman measurements.

Two sets of Raman measurements were done using the printed cuvettes. Firstly, three *G. sulfurreducens* mutants (PCA, KN400 and ΔPilA) were grown in serum bottles using NBAF growth media containing a stoichiometric excess of acetate (approximately 20 mM acetate and 32 mM fumarate) to ensure that growth is limited by the electron acceptor [14]. Once sufficient biomass was obtained, samples were taken in an anaerobic box at regular intervals during the growth stage (exponential and stationary phase). After initial measurements, the kinetic effect of fumarate addition was studied by addition of additional fumarate (approx. 5 mM). This is illustrated in Figure 10.

Secondly, in order to obtain controllable conditions, biomass was grown using a chemo-stat with an excess of electron donor (25 mM acetate and 40 mM fumarate), similar to the study of Nunez et al. [13,31]. Three 25 mL samples were then taken, centrifuged and the cells of each were re-suspended in 4 mL 50 mM phosphate buffer (pH 7). The samples were prepared in an anaerobic box to prevent the oxidation of the heme groups. No electron donor was added to the re-suspended cells.

**Figure 11:**
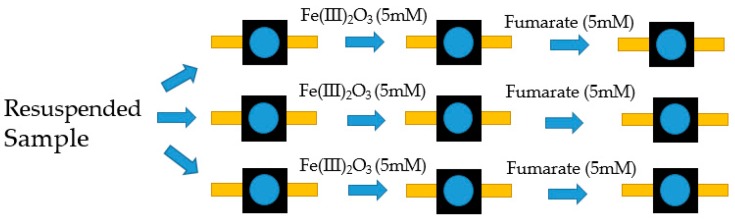
Schematic of the sampling process used for Chemostat grown biomass Raman measurements.

After initial measurements, 5 mM Fe(III)oxide was added to the samples, in order to act as an extracellular electron acceptor, and measured again. Finally, additional fumarate (approx. 5 mM) was added, which acts as an electron acceptor that can be incorporated into the cell, and measured again (a schematic is shown in Figure 11).

### 3.4. Microbial Fuel Cell Set Up and Raman Measurements

The cell was sterilized by passing ethanol through the cell for approximately an hour followed by a rinse with 500 mL of sterile milli-Q water. After sterilization the cell was connected to two 250 mL bottles filled with freshwater medium with 20 mM acetate and 40 mM fumarate on the anode side. The media was recirculated through the reactor and the bottles were sparged continuously with 20:80 CO_2_/N_2_ to maintain anaerobic conditions. The cells were poised at 300 mV (using a MultiEmStat^+^ potentiostat, PalmSens, Netherlands) and the anode side was inoculated with 10% of the inoculum. At the point of maximum current production, the anode side bottle was switched to a continuous feed of freshwater media, containing only 10 mM acetate, at a flow rate of 0.5 mL/min. The feed was maintained for approximately 2–4 weeks before the biofilm was clearly visible as a pink layer.

During measurements, the MFC stack was initially poised at 300 mV, and then lowered to −150 mV and increased back to 300 mV, in 150 mV intervals, taking two measurements at each poised voltage. The voltage was then changed directly to −300 mV, back to 300 mV and finally the potentiostat was disconnected.

### 3.5. Analytical Methods

The Raman spectra, taken using the printed cuvettes, were collected with a Ramanscope III spectrometer in combination with a SENTERRA module allowing dispersive Raman microanalysis. The system was equipped with a 532 nm excitation laser for use in the standard normal incidence sampling geometry. The data were accumulated in the range from 30 to 1550 cm^−1^ using an incident laser power of about 20 mW. An integration time of 10 s with 10 co-addition scans was used to acquire the spectra.

Raman spectra for the stack measurements were collected using an inverted microscope (Olympus IX71, Tokyo, Japan) equipped with 532 nm excitation DPPS laser (Laserglow Technologies, Toronto, Canada) and coupled to a Raman spectrometer, Shamrock 303i (Andor Technology, Belfast, UK). The laser was operated at approximately 15 mW prior to the microscope objective, for 120 s.

The raw Raman spectra was processed in the following way: First the cosmic rays were removed using an automated tool. [43] A chromatogram baseline estimation and denoising filter using sparsity was used to remove fluorescence and background noise [44]. Finally, a Savitzky-Golay de-noising filter was used to smooth the signal [45]. The smoothed curve was then integrated over a fixed window of 22 cm^−1^ using Matlab R2018 to obtain the area below each peak. The peaks were compared to results from Virdis et al. and was found to have nearly identical peaks [34]. Four strong bands were observed at 744, 1131, 1317 and 1587 cm^−1^ which can be ascribed to the excitation of the heme groups of cytochrome c that are prevalent in the biofilms [29,46].

Biomass concentrations were determined using the optical density at 600 nm. The concentration of organic acids and sugars were determined using a Perkin-Elmer HPLC system with a Series 200 refractive index detector, as previously described [47].

## 4. Conclusions

The 3D printed cuvette functioned well throughout all Raman measurements using isolated suspended cells from three strains. Raman spectra were obtained for the KN400, ΔpilA and PCA strains in suspended cultures, showing that the values of the Raman peak areas can be used as an indicator of the cytochrome redox state of the cells. This is further stressed by the observation of lower Raman peak areas when using KN400, due to the presence of less OMCs when compared to PCA. Furthermore, a higher capacity for electron transport in the wild type when compared to ΔpilA was also observed. It was also shown that PilA is not required for external electron transfer to occur.

An anaerobic fluidic system enabling in-situ Raman measurements was designed and applied successfully to monitor and characterize *G. sulfurreducens* biofilms during electricity generation. The redox state of the cytochromes, monitored by the Raman peak area, could be modulated by applying voltage to the electrodes and this is correlated with the modulation of current flowing between the cytochromes and the electrode. Through monitoring an MFC with a ∆OmcS biofilm it was also shown that OmcS is not required for effective electron transfer to an external electrode.

Raman microscopy was shown to be capable of providing biochemical information, i.e., the redox state of C-type cytochromes, without the need to interfere with the operation of the MFC, the removal of the electrode or the staining of the biofilm. Therefore, the biofilm and suspended cultures could be studied on-line in completely anaerobic conditions. Since these cytochromes are an integral part of the electron transport chain, Raman measurements can, for example, be used to give information on electron transfer to external electrodes. Hence, this study shows that Raman measurements can serve as a useful tool for elucidating the mechanisms involved in the electron transport chain, as well as to analyze the metabolic state of active biofilms.
